# Protective effects of *Exocarpium Citri Grandis* against sepsis-induced acute lung injury via PANoptosis inhibition

**DOI:** 10.3389/fnut.2025.1661404

**Published:** 2025-12-22

**Authors:** Zaibin Xu, Kongyan Wang, Huiyu Hu, Yan Chen, Yi Qiu, Jiazhong Cai, Weirong Li, Zhuohui Luo, Hang Li, Jiawen Huang

**Affiliations:** 1 Science and Technology Innovation Center, Guangzhou University of Chinese Medicine, Guangzhou, China; 2 School of Chinese Materia Medica, Guangdong Yunfu Vocational College of Chinese Medicine, Yunfu, China; 3 Research Center for Drug Safety Evaluation of Hainan Province, Hainan Medical University, Haikou, China; 4 Hainan Pharmaceutical Research and Development Science Park, Haikou, China; 5 Central Lab, Shenzhen Bao’an Chinese Medicine Hospital, Guangzhou University of Chinese Medicine, Shenzhen, China

**Keywords:** sepsis, acute lung injury, *Exocarpium Citri Grandis*, PANoptosis, inflammation

## Abstract

Sepsis-induced acute lung injury (ALI) is a life-threatening condition with high mortality, driven by dysregulated inflammation and programmed cell death. *Exocarpium Citri Grandis* (ECG), which refers to the dried outer rind of the Rutaceae plant *Citrus grandis* ‘Tomentosa’ or *Citrus grandis* (L.) Osbeck, has a history of use in treating pulmonary inflammatory diseases, yet its mechanism of action against sepsis-induced ALI remains unexplored. In this study, we investigated the therapeutic potential of ECG in a murine model of sepsis induced by cecal ligation and puncture (CLP). This study reveals that ECG significantly reduces the levels of IL-1β, IL-6, and TNF-*α* in both serum, bronchoalveolar lavage fluid (BALF) and in lung tissue. Additionally, ECG downregulates the abnormal expression of chemokine genes. Molecular dynamics simulations revealed that Naringin and Neohesperidin, active compounds of ECG, form stable complexes with the PANoptosis-related proteins ZBP1 and RIPK1. The protective effect of ECG was mediated through the simultaneous targeting of multiple programmed cell death pathways, as it inhibited NLRP3/Caspase-1/GSDMD-driven pyroptosis, suppressed Bcl-2/Bax/Caspase-3-dependent apoptosis, and attenuated ZBP1/MLKL/RIPK1-induced necroptosis. Our study identifies ECG as a multi-target therapeutic agent that mitigates sepsis-induced ALI primarily through the inhibition of PANoptosis, providing a mechanistic foundation for its potential development as a functional food or dietary intervention strategy.

## 1 Introduction

Sepsis is a life-threatening syndrome characterized by organ dysfunction due to a dysregulated host response to infection, accounting for nearly 20% of global mortality (1, 2). The lungs are among the most vulnerable organs, with acute lung injury (ALI) frequently developing in the early stages and significantly contributing to patient mortality (3, 4). Despite advances in understanding the inflammatory basis of sepsis-induced ALI, effective therapeutic strategies targeting specific cell death pathways remain limited (5).

PANoptosis is a unique inflammatory programmed cell death pathway which integrates key features of pyroptosis, apoptosis, and necroptosis, and recent evidence highlights its critical role in driving tissue damage and immune dysregulation in sepsis (6, 7). Activation of the PANoptosis pathway leads to the release of damage-associated molecular patterns (DAMPs), which further amplify inflammatory cascades and recruit immune cells, establishing a vicious cycle of injury (8, 9). Although pharmacological inhibition of PANoptosis has shown promise in preclinical models (10–12), the potential of dietary or natural compounds to modulate this pathway in sepsis-induced ALI remains largely unexplored.

*Exocarpium Citri Grandis* (ECG), the dried outer peel of *Citrus grandis*, has been traditionally used for treating pulmonary inflammatory conditions (13, 14). Previous studies from our group have demonstrated that ECG exerts anti-inflammatory and anti-apoptotic effects in models of chronic bronchitis and LPS-induced ALI, involving mechanisms such as TLR4/MyD88/NF-κB and Nrf2/GPX4 signaling (15). However, whether ECG can attenuate sepsis-induced ALI via regulation of PANoptosis has not been investigated. Given the multi-component, multi-target nature of ECG, we hypothesized that it may confer protection against sepsis-induced ALI by concurrently modulating multiple cell death pathways within the PANoptosis framework. To test this, we employed a murine model of polymicrobial sepsis induced by cecal ligation and puncture (CLP), a gold standard model that replicates the hyperinflammatory and hypodynamic phases of clinical sepsis (16–18). In our model, the cecum was ligated at the distal third and punctured twice with a 18-gage needle to induce a moderate-grade sepsis, followed by fluid resuscitation to increase clinical relevance and survival (19). Using this model, combined with molecular and cellular assays, this study aims to elucidate the role of ECG in inhibiting PANoptosis and its potential as a functional food intervention for sepsis-associated lung injury.

## 2 Materials and methods

### 2.1 Chemicals and reagents

Commercial ELISA kits were obtained from Thermo (Thermo Fisher Scientific Inc., United States). Necroptosis Inducer Kit (Cat. No. C1058S) and the Annexin V-FITC Apoptosis Detection Kit (Cat. No. C1062M), both obtained from Beyotime Biotechnology (Beyotime Biotechnology Inc., China). Anti-TLR4, anti-MYD88 and anti-p-RIPK1 were purchased from Proteintech (Proteintech Group, Inc., United States). Anti-NF–κB p65, Anti-NF–κB p-p65 (S536), anti-NLRP3, antiCaspase1, anti-ASC, anti-NEK7, anti-GSDMD, anti-IL-1*β*, anti-IL-18 and anti-β-Actin were obtained from ImmunoWay (ImmunoWay Biotechnology Company, USA). Anti-IkBa, anti-p-IkBa (S36), anti-ICAM1, anti-COX2, anti-INOS, anti-MLKL, anti-p-MLKL (ser358) and anti-RIPK1 were provided by Affinity (Affinity Biosciences, China). Anti-ZBP1 was obtained from Santa Cruz (Santa Cruz Biotechnology, Inc. United States). Unless specifically mentioned, the remaining substances were obtained from Sigma-Aldrich.

### 2.2 ECG preparation

Following established protocols (20), the ECG herbal material (Batch No. Z46-20220901), which served as the monarch medicine in Honz Pharmaceutical Co., Ltd.’s “Zhike Juhong Granules,” was extracted under optimized conditions. Specifically, 100.1 g of ECG herb underwent reflux extraction for 1 h using an eightfold volume of 50% aqueous ethanol (v/v). The mixture was subsequently filtered through gauze to obtain the first filtrate. The remaining residue was subjected to a second round of reflux extraction with a sixfold volume of 50% ethanol-water solution (v/v) for an additional hour, followed by filtration to yield the second filtrate. Both filtrates were then combined, concentrated under reduced pressure, and finally lyophilized, resulting in a solid extract with a yield of 41.61%. The lyophilized product was further characterized by UPLC-Q/TOF-MS/MS analysis (see Supplementary Figure 1; Supplementary Table 1).

### 2.3 Animals

Male C57BL/6 mice (8 weeks old, weighing 20.0–22.0 g) were purchased from the Guangdong Medical Experimental Animal Center (Guangzhou, China). All mice were housed under SPF conditions at a controlled temperature (22–26 °C) with a 12-h light/dark cycle, and provided with adequate food and water ad libitum. All experimental protocols have been approved by the Ethics Committee of Guangzhou University of Traditional Chinese Medicine, with approval number 20240122005.

### 2.4 CLP model and drug treatment

Mice were randomly assigned to the following five groups (n = 6/group): Control, CLP, CLP + ECG (25 mg/kg), CLP + ECG (50 mg/kg), and CLP + ECG (100 mg/kg). In brief, following a 7-day prophylactic regimen of either ECG or vehicle, anesthesia was induced in the mice via intraperitoneal administration of sodium pentobarbital (50 mg/kg). Under sterile conditions, a 1 cm midline abdominal incision was made to access the cecum. The distal third of the cecum was securely ligated using a 3–0 silk suture, taking care to avoid complete intestinal obstruction. This ligated segment was then perforated once with a 18-gage needle. A minimal quantity of fecal material was expressed from the perforation sites to confirm and maintain patency. Subsequently, the cecum was carefully repositioned into the abdominal cavity. Closure of the peritoneum and skin was performed in layers using a 5–0 absorbable suture and interrupted non-absorbable sutures, respectively. Control animals in the sham group were subjected to identical surgical procedures, including laparotomy and cecal exteriorization, but did not undergo cecal ligation or puncture. Following surgery, all mice received supportive care consisting of a 1 mL subcutaneous injection of pre-warmed sterile saline for resuscitation and were maintained on a heating pad until complete recovery from anesthesia.

### 2.5 Survival analysis

Male C57BL/6 mice (8 weeks old, weighing 20.0–22.0 g) were randomly assigned to one of five groups (*n* = 10/group): Control, CLP, CLP + ECG (25 mg/kg), CLP + ECG (50 mg/kg), and CLP + ECG (100 mg/kg). A 72-h survival analysis was conducted on CLP-induced septic mice following a 7-day prophylactic regimen of either ECG or vehicle, with survival data recorded at 12-h intervals.

### 2.6 BALF collection

Expose the trachea of mice after death, insert a sterile indwelling needle of size 22 horizontally into the trachea, and ligate it below the needle insertion point. Then, wash the bronchoalveolar space three times with 1.2 mL PBS and collect BALF (approximately 0.8–1.0 mL).

### 2.7 Giemsa staining

After collecting the supernatant of BALF, centrifuge to obtain the cells contained therein. Then, according to the instructions of the kit, use Wright Giemsa staining solution (Beyotime Biotechnology, China) to stain the cells. The stained image was scanned using a digital scanner (Kong Feng Bioinformatics Technology Co., Ltd., Ningbo, China).

### 2.8 IL1β, IL6 and TNF-*α* measurement

Serum, BALF and lung tissue levels of IL1β, IL6, and TNF-α were determined according to the instructions of the mouse uncoated ELISA kits with plates (eBioscience, United States).

### 2.9 H&E staining

The lung tissue was fixed with 4% paraformaldehyde, and paraffin embedded tissue sections were prepared, followed by the standard procedure of hematoxylin eosin (HE) staining. Images were acquired using a digital pathological slice scanner (Kong Feng Bioinformatics Technology Co., Ltd., Ningbo, China). Observe histopathological changes such as pulmonary edema, bleeding, and inflammatory cell infiltration. We then employed a well-established scoring system, utilizing the acute lung injury histopathological scoring criteria defined by the American Thoracic Society (21, 22). The assessment was based on four key parameters: alveolar congestion, hemorrhage, infiltration or accumulation of neutrophils in the alveolar spaces or vascular walls, and alveolar wall thickening/hyaline membrane formation. Each parameter was graded on a 5-point scale from 0 to 4, defined as follows: 0 (absent/within normal limits), 1 (minimal, involving <25% of the field), 2 (mild, involving 25–50% of the field), 3 (moderate, involving 50–75% of the field), and 4 (severe, with diffuse involvement >75% of the field). A cumulative lung injury score, ranging from 0 to 16, was derived by summing the individual scores from all four categories.

### 2.10 Western blotting

The proteins in lung tissue were lysed and extracted by RIPA buffer (Beyotime Biotechnology, China) containing inhibitors. Quantitatively determine the concentration of extracted protein using BCA (Beyotime Biotechnology, CHN) protein detection kit. Separate an equal amount of total protein using 10% SDS-PAGE and transfer it onto a PVDF membrane (pore size: 0.22 μm or 0.45 μm, Millipore). Seal the membrane with western blot rapid blocking buffer (Termo, United States) at room temperature for 10 min and incubate overnight with specific primary antibody at 4 °C. Subsequently, the secondary antibody coupled with horseradish peroxidase was bound at room temperature for 1 h, and the protein bands were observed using ECL chemiluminescence reagent(Guangdong Prochem Biotechnology Co., Ltd., Guangdong, China) using a Molecular Imager® System (BIO-RAD, CA, United States). ImageJ (NIH, Bethesda, MD, United States) was used to quantify the expression.

### 2.11 Rt-PCR

Total RNA was extracted from lung tissues using TRIzol reagent (Invitrogen, Carlsbad, CA, United States) according to the manufacturer’s instructions. RNA concentration and purity were determined spectrophotometrically, with all samples having an A260/A280 ratio between 1.8 and 2.0. Subsequently, 1 μg of total RNA was reverse-transcribed into cDNA using the PrimeScript™ RT Master Mix (Takara Biomedical Technology Co. Ltd., Dalian, China). Quantitative PCR was performed on a CFX384 Touch™ Real-Time PCR Detection System (Bio-Rad Laboratories (Shanghai) Co., Ltd.) using TB Green® Premix Ex Taq™ II (Takara Biomedical Technology Co. Ltd., Dalian, China). The reaction mixture (25 μL) contained 12.5 μL of TB Green Premix, 1.0 μL each of forward and reverse primers, 2 μL of cDNA template, and 8.5 μL of nuclease-free water. The thermal cycling conditions were as follows: initial denaturation at 95 °C for 30 s, followed by 40 cycles of 95 °C for 5 s and 60 °C for 30 s. A melt curve analysis was performed at the end of each run to confirm amplification specificity. The expression of target genes was normalized to the housekeeping gene Actb (*β*-actin) and calculated using the 2^(-ΔΔCt) method. All primer sequences are listed in Supplementary Table 2.

### 2.12 Immunofluorescence

Wash lung tissue with pre cooled PBS to remove impurities such as blood. Cut the tissue into small pieces and fix them with paraformaldehyde for 2–4 h to maintain tissue morphology and antigen activity. The fixed tissue is subjected to dehydration, transparency, wax immersion, and embedding treatment. Then cut the embedded tissue into thin slices with a thickness of about 4–6 *μ* m. Slices were deparaffinized to water and antigen repair was performed using antigen repair solution. Add primary antibody dropwise and incubate overnight at 4 °C in a wet box. After washing with PBS, add the corresponding fluorescently labeled secondary antibody dropwise and incubate at room temperature in the dark for 1–2 h. Finally, seal the tissue with anti fluorescence quenching sealing agent, Images were taken of each slice using a digital pathological slice scanner (Kong Feng Bioinformatics Technology Co., Ltd., Ningbo, China).

### 2.13 RNA sequencing

Total RNA was isolated from lung tissues (*n* = 3 per group) as described in section 2.10. RNA purity and concentration were assessed using a NanoDrop 2000 spectrophotometer (Thermo Scientific, United States), and RNA integrity was evaluated with an Agilent 2,100 Bioanalyzer (Agilent Technologies, Santa Clara, CA, USA). Sequencing libraries were constructed with the VAHTS Universal V5 RNA-seq Library Prep Kit (Vazyme Biotech, Nanjing, China) and sequenced on an Illumina NovaSeq 6,000 platform (Illumina, San Diego, CA, United States) to generate 150 bp paired-end reads. For bioinformatics analysis, raw data were processed with fastp to obtain clean reads, which were then aligned to a reference genome using HISAT2 for gene expression quantification. Differential expression analysis was performed with DESeq2 (|fold change| > 2 and q-value < 0.05), followed by functional enrichment analysis (GO, KEGG, etc.) and visualization in R (v 3.2.0).

### 2.14 Cells culture

RAW264.7 cells were maintained in RPMI 1640 medium supplemented with 10% fetal bovine serum (FBS), 100 U/mL penicillin, and 100 μg/mL streptomycin. Cultures were grown at 37 °C in a humidified 5% CO₂ atmosphere. Prior to experimental procedures, cells were plated on culture vessels and allowed to adhere for 24 h. For treatments, cells underwent pre-exposure to ECG at varying concentrations (25, 50, or 100 μg/mL) for 1 h, followed by stimulation with 0.5 μg/mL LPS. After 24 h of LPS exposure, either cellular material or conditioned media were harvested for downstream assays.

### 2.15 Detection of apoptosis and necrosis

Apoptosis and necroptosis in LPS-induced RAW264.7 cells were assessed using commercial detection kits, specifically the Necroptosis Inducer Kit and the Annexin V-FITC Apoptosis Detection Kit. Briefly, cells were treated with the necroptosis inducer at a 1:250 dilution for 8 h. After incubation, the culture medium was removed, and the cells were washed once with PBS. Subsequently, the cells were resuspended in 195 μL of Annexin V-FITC binding buffer, followed by the addition of 5 μL of Annexin V-FITC and 10 μL of propidium iodide (PI) staining solution. The cell suspension was gently mixed and incubated at room temperature (20–25 °C) in the dark for 10–20 min. Following incubation, the samples were placed on ice to halt the staining process. Fluorescence signals were visualized immediately under a fluorescence microscope, with Annexin V-FITC and PI exhibiting green and red fluorescence, respectively.

### 2.16 Molecular dynamics simulations

To investigate the molecular mechanisms of ZBP1 and RIPK1 binding with Naringin and Neohesperidin, molecular dynamics simulations were performed using Gromacs 2020. The protein-ligand complexes were modeled with AMBER99SB-ILDN for proteins and GAFF for ligands, with ligand topologies generated by sobtop using RESP charges. The system was solvated in TIP3P water with 1.0 nm buffer distance and neutralized by Na^+^/Cl^−^ ions. The simulation protocol included four stages: (1) Dual-step energy minimization (10,000 steps total) with initial heavy-atom constraints followed by full-system optimization. (2) Gradual heating to 300 K over 50 ps. (3) 50 ps NPT equilibration. (4) 100 ns production MD under NPT ensemble with trajectories saved every 10 ps. Binding free energies were calculated using gmx_MMPBSA.

### 2.17 Statistical analysis

All statistical analyses and graphing were performed using SPSS 27.0 and GraphPad Prism software version 9.0 (San Diego, CA, United States). Data are presented as the mean ± SEM from at least three independent experiments. For data sets that satisfied the assumptions of normality and homogeneity of variances, comparisons between groups were made using one-way ANOVA. For data that were normally distributed but had unequal variances, Dunnett’s T2 test was applied. If the data did not meet the normality assumption, non-parametric tests were employed. Differences were regarded as statistically significant when *p* < 0.05.

## 3 Results

### 3.1 ECG improves the infiltration and exudation of pro-inflammatory cells in sepsis induced ALI mice

**Figure 1:**
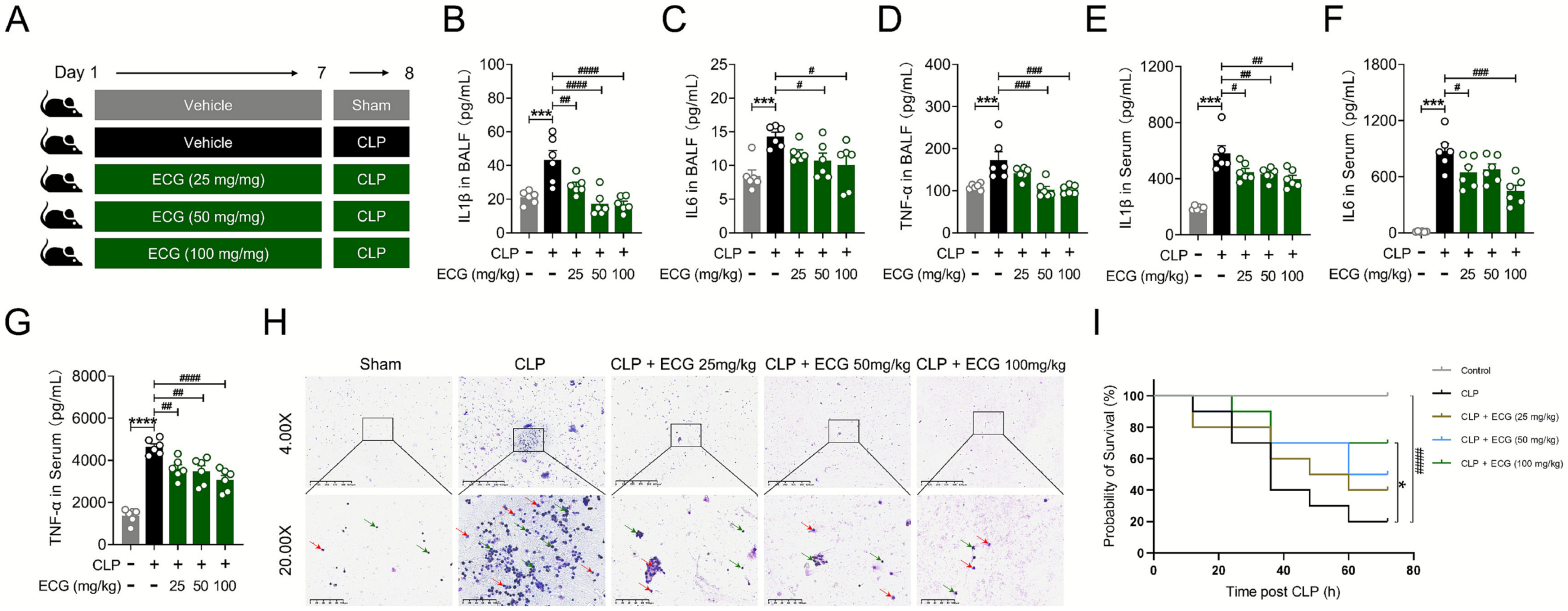
ECG reduced the infiltration and exudation of pro-inflammatory cells in sepsis induced ALI mice. **(A)** *In vivo* experimental process. **(B–G)** IL1β, IL6, and TNF-*α* levels in BALF and Serum (*n* = 6). **(H)** Representative images of Giemsa staining. Scale bars: 625 μm and 100 μm. Neutrophils in light purple-red (arrows: green) and macrophages in blue-purple (arrows: red). **(I)** Shows the survival and death situation of CLP-induced sepsis mice (*n* = 10). Data are presented as mean ± SEM. Compared to the control group, ***p < 0.001, and ****p < 0.0001. Compared to the CLP group, ^#^*p* < 0.05, ^##^*p* < 0.01, ^###^*p* < 0.001, and ^####^*p* < 0.0001.

To delve deeper into the impact of ECG on sepsis-induced ALI, we established a CLP-induced sepsis mouse model (Figure 1A), and ELISA kits were used to detect the levels of inflammatory cytokines in BALF and serum of mice in each group. The findings revealed a significant elevation in the levels of interleukin-1β (IL-1β), interleukin-6 (IL-6), and tumor necrosis factor-*α* (TNF-α) in both BALF and serum of sepsis-induced ALI mice. ECG pretreatment significantly attenuated this inflammatory response in a clear dose-dependent manner. The reduction of pro-inflammatory cytokines was most pronounced in the CLP + ECG (100 mg/kg) group, which showed levels approaching those of the sham controls, while the CLP + ECG (50 mg/kg) and CLP + ECG (25 mg/kg) groups exhibited intermediate and milder effects, respectively (Figures 1B–G). Additionally, Giemsa staining of BALF revealed abnormal inflammatory cell aggregation in the CLP group, a pathological alteration that was markedly reversed by ECG treatment. The aggregated cells included neutrophils, appearing light purple-red, and macrophages, which stained blue-purple. This protective effect against cellular infiltration was dose-dependent, with the most substantial reduction observed in the CLP + ECG (100 mg/kg) group (Figure 1H). Simultaneously, we conducted a 72-h survival rate monitoring experiment in CLP-induced septic mice (*n* = 10) (23–25). The results showed a 100% survival rate in the Control group, compared to only 20% in the CLP model group. In contrast, ECG treatment resulted in a dose-dependent improvement in mouse survival (Figure 1I). These experimental results suggest that ECG significantly improves the infiltration and exudation of pro-inflammatory cells in lung tissue of ALI mice.

### 3.2 ECG improves inflammation in sepsis induced ALI mice

**Figure 2:**
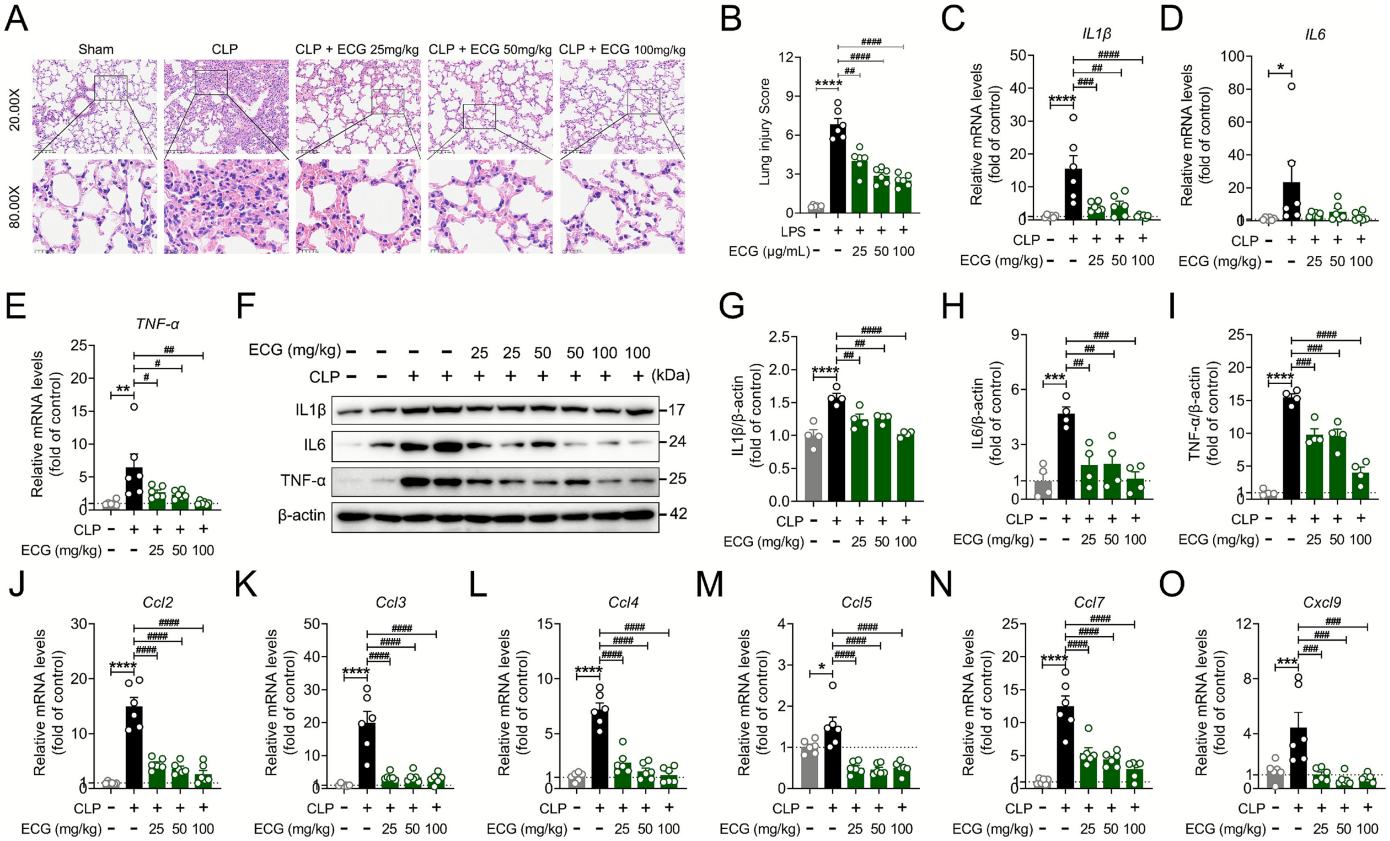
ECG reduces inflammatory response in sepsis induced ALI mice. **(A)** Representative images of H&E staining. Scale bars: 100 μm and 25 μm. **(B)** Lung injury score (*n* = 6). **(C–E)** mRNA levels of inflammatory factors genes, including *IL-1β, IL-6,* and *TNF-α* (*n* = 6). **(F)** Western blotting analysis of IL-1β, IL-6, and TNF-α proteins in lung tissues. **(G-I)** Relative intensity of IL-1β, IL-6, and TNF-α (*n* = 4). **(J-O)** mRNA levels of chemokine-related genes, including *Ccl2*, *Ccl3*, *Ccl4*, *Ccl5*, *Ccl7*, *and Cxcl9* (*n* = 6). Data are presented as mean ± SEM. Compared to the control group, *p < 0.05, **p < 0.01, ***p < 0.001, and ****p < 0.0001. Compared to the CLP group, ^#^*p* < 0.05, ^##^*p* < 0.01, ^###^*p* < 0.001, and ^####^*p* < 0.0001.

The infiltration and exudation of a large number of inflammatory cells are often closely linked to the onset of rapid inflammatory reactions in the body (26–28). Therefore, we conducted further research on the lung tissue of mice. The pathological examination of lung tissue revealed abnormal inflammatory cell infiltration and significant damage to the lung tissue structure in the CLP group (Figure 2A). And the histopathological scoring results clearly demonstrated that ECG pretreatment mitigated this damage in a dose-dependent manner (Figure 2B). The CLP + ECG (25 mg/kg) group showed a modest reduction in cellular infiltration, while the CLP + ECG (50 mg/kg) and CLP + ECG (100 mg/kg) groups exhibited progressively greater protection, with near-normal lung architecture observed at the highest dose. As anticipated, the mRNA expression of IL-1β, IL-6, and TNF-*α* in the lung tissue of the CLP group was abnormally elevated, but it significantly decreased following ECG administration (Figures 2C–E). Simultaneously, the results of immunoblotting experiments indicated that the expression of IL-1β, IL-6, and TNF-α proteins in both the CLP group and the treatment group closely aligned with the aforementioned experimental findings (Figures 2F–I). Furthermore, our qPCR experiment revealed that the mRNA expression of various chemokines, including Ccl2, Ccl3, Ccl4, Ccl5, Ccl7, and Cxcl9, was abnormally high in CLP-induced ALI mice. Notably, the suppression of this chemokine cascade was most effective at the highest dose of 100 mg/kg (Figures 2J–O). This suggests that ECG exerts an inhibitory effect on the inflammatory response in sepsis-induced ALI mice.

### 3.3 ECG inhibited NLRP3/Caspase-1/GSDMD pathwayin in sepsis induced ALI mice

**Figure 3:**
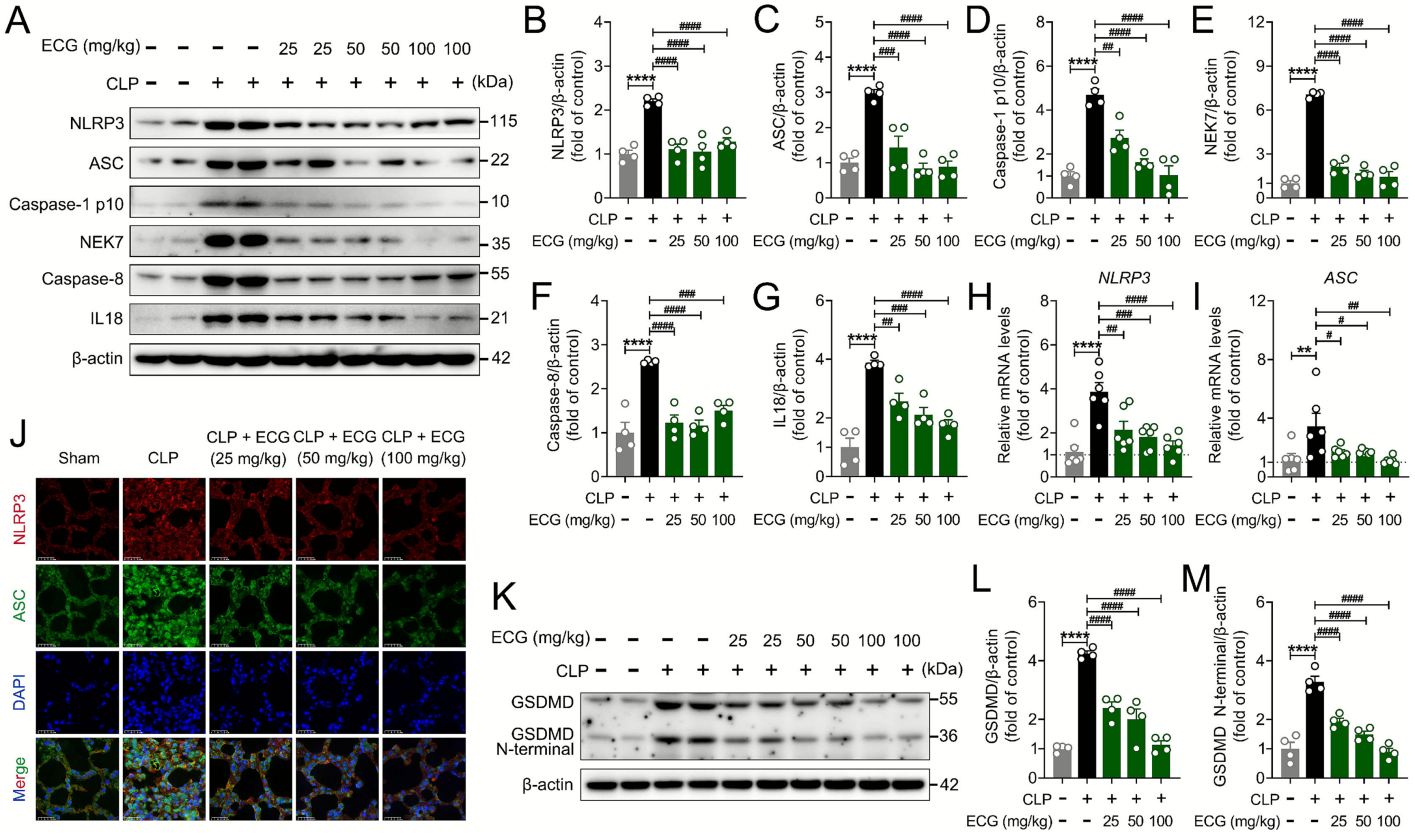
ECG inhibited NLRP3/Caspase-1/GSDMD pathwayin in sepsis induced ALI mice. **(A)** Western blotting analysis of NLRP3, ASC, Caspase-1 p10, NEK7, Caspase-8, and IL18 proteins in lung tissues. **(B–G)** Relative intensity of these proteins (*n* = 4). **(H,I)** mRNA levels of *NLRP3* and *ASC* genes (*n* = 6). **(J)** Representative immunofluorescence images of NLRP3-positive, and ASC-positive in lung tissues. Scale bars: 25 μm. **(K)** Western blotting analysis of GSDMD and GSDMD N-terminal proteins proteins in lung tissues. **(L,M)** Relative intensity of GSDMD and GSDMD N-terminal (*n* = 4). Data are presented as mean ± SEM. Compared to the control group, **p < 0.01, and ****p < 0.0001. Compared to the CLP group, ^#^*p* < 0.05, ^##^*p* < 0.01, ^###^*p* < 0.001, and ^####^*p* < 0.0001.

To explore the molecular mechanism by which ECG improves sepsis-induced ALI, we evaluated the impact of ECG intervention on NLRP3 signaling transduction. During the process of clearing pathogens or foreign objects, infiltrating inflammatory cells release more inflammatory mediators and reactive oxygen species, which can further activate NLRP3 inflammasomes, thereby stimulating the secretion of IL1β and IL18 and triggering the pyroptosis of inflammatory cells (29). As expected, immunoblotting experiments confirmed that ECG significantly reduced the expression of NLRP3, ASC, Caspase-1 p10, NEK7, Caspase-8, and IL18 proteins. ECG pretreatment potently suppressed the expression of these proteins in a clear dose-dependent manner. The most profound inhibition was consistently observed in the CLP + ECG (100 mg/kg) group, which often restored protein levels to near-baseline, while the CLP + ECG (50 mg/kg) and CLP + ECG (25 mg/kg) groups showed intermediate and modest effects, respectively (Figures 3A–G). Consistent with this, the mRNA expression of inflammasome-related genes NLRP3 and ASC was also downregulated after ECG treatment (Figures 3H,I). Simultaneously, immunofluorescence staining revealed that ECG intervention significantly decreased the expression of NLRP3 and ASC (Figure 3J), aligning closely with the aforementioned observations. Additionally, activated caspase-1 is a crucial enzyme that cleaves GSDMD, releasing its N-terminal domain and binding to the cell membrane, thereby triggering cell pyroptosis. Critically, this inhibition of pyroptotic execution also exhibited a dose–response relationship, with the most effective of GSDMD cleavage achieved at the highest dose of ECG (Figures 3K–M). In summary, ECG exerts an ameliorative effect on sepsis-induced ALI by inhibiting the NLRP3/Caspase-1/GSDMD signaling pathway.

### 3.4 ECG inhibits sepsis induced apoptosis in ALI mice

**Figure 4:**
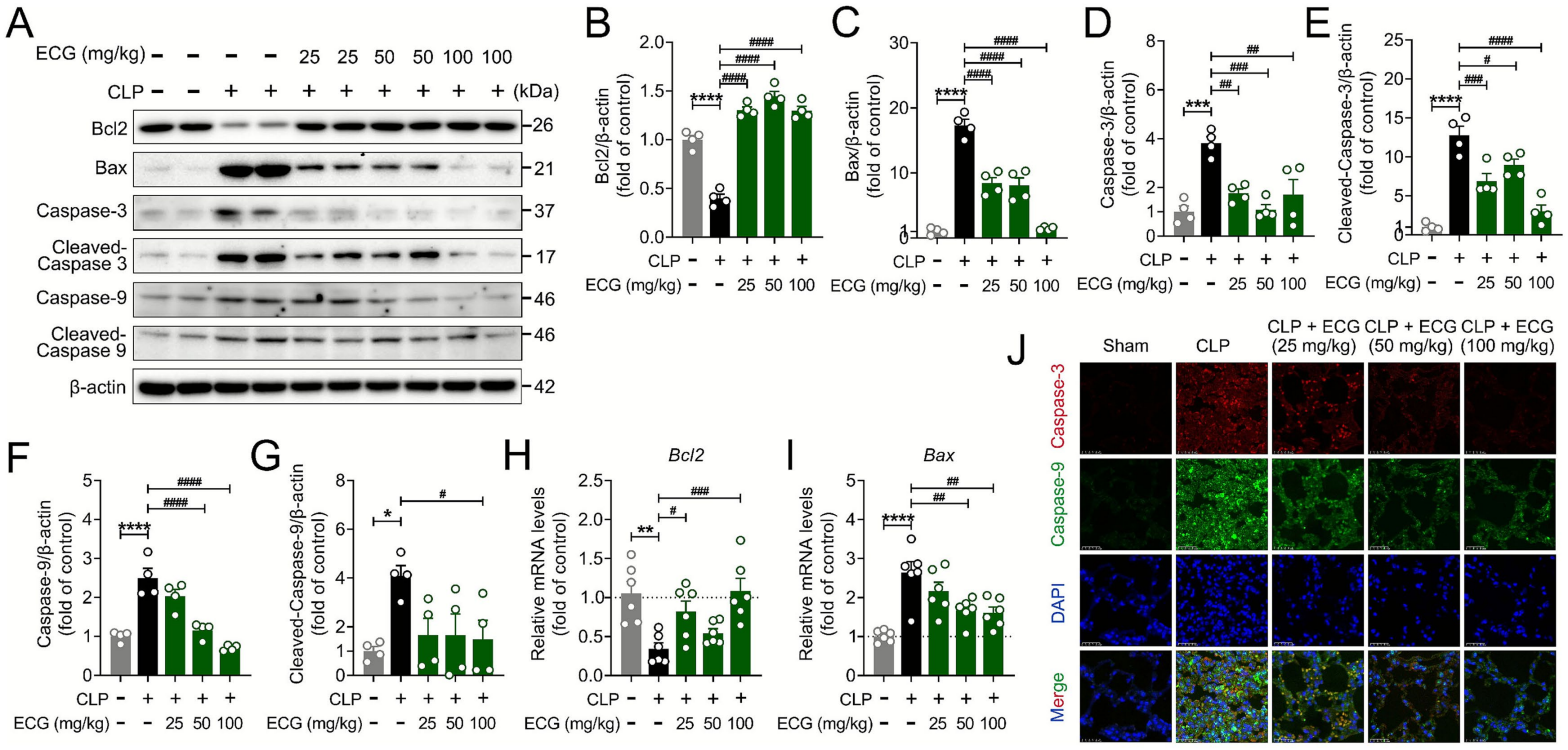
ECG inhibits sepsis induced apoptosis in ALI mice. **(A)** Western blotting analysis of Bcl2, Bax, Caspase3, Cleaved-Caspase3, Caspase9, and Cleaved-Caspase9 proteins in lung tissues. **(B–G)** Relative intensity of these proteins (*n* = 4). **(H,I)** mRNA level of *Bcl2* and *Bax* (*n* = 6). **(J)** Representative immunofluorescence images of Caspase3-positive, and Caspase9-positive in lung tissues. Scale bars: 25 μm. Data are presented as mean ± SEM. Compared to the control group, *p < 0.05, **p < 0.01, ***p < 0.001, and ****p < 0.0001. Compared to the CLP group, ^#^*p* < 0.05, ^##^*p* < 0.01, ^###^*p* < 0.001, and ^####^*p* < 0.0001.

Recent studies have revealed a close association between cell pyroptosis and cell apoptosis. During the process of cell apoptosis, the integrity of the cell membrane and the formation of inflammasomes influence the progression of apoptosis (30). To delve deeper into the pharmacological mechanism of ECG, we employed immunoblotting, real-time quantitative PCR, and fluorescence immunoassay to examine the impact of ECG on sepsis-induced apoptosis in ALI mice. The findings indicated that sepsis-induced ALI mice exhibited pronounced cell apoptosis. Notably, ECG pretreatment markedly attenuated this apoptotic response, with the degree of protection being contingent upon the administered dose. Furthermore, ECG effectively upregulated the expression of the anti-apoptotic protein Bcl-2 and downregulated the expression of pro-apoptotic proteins, including Bax, Caspase3, Cleaved-Caspase3, Caspase9, and Cleaved-Caspase9 (Figures 4A–G). Additionally, PCR data revealed an upregulation in the mRNA expression of the apoptosis gene Bcl2 and a downregulation in Bax mRNA expression in the ECG-treated group of mice (Figures 4H,I). Aligning with these results, immunofluorescence assay demonstrated significantly elevated expression of Caspase-3 and Caspase-9 in the CLP group. This elevated expression was notably reduced by ECG pretreatment, with the most significant decrease observed in the CLP + ECG (100 mg/kg) group (Figure 4J). This suggests that ECG plays a pivotal role in sepsis-induced ALI mice by suppressing cell apoptosis.

### 3.5 ECG protects sepsis induced ALI mice by inhibiting the ZBP1/MLKL/RIPK1 signaling pathway

**Figure 5:**
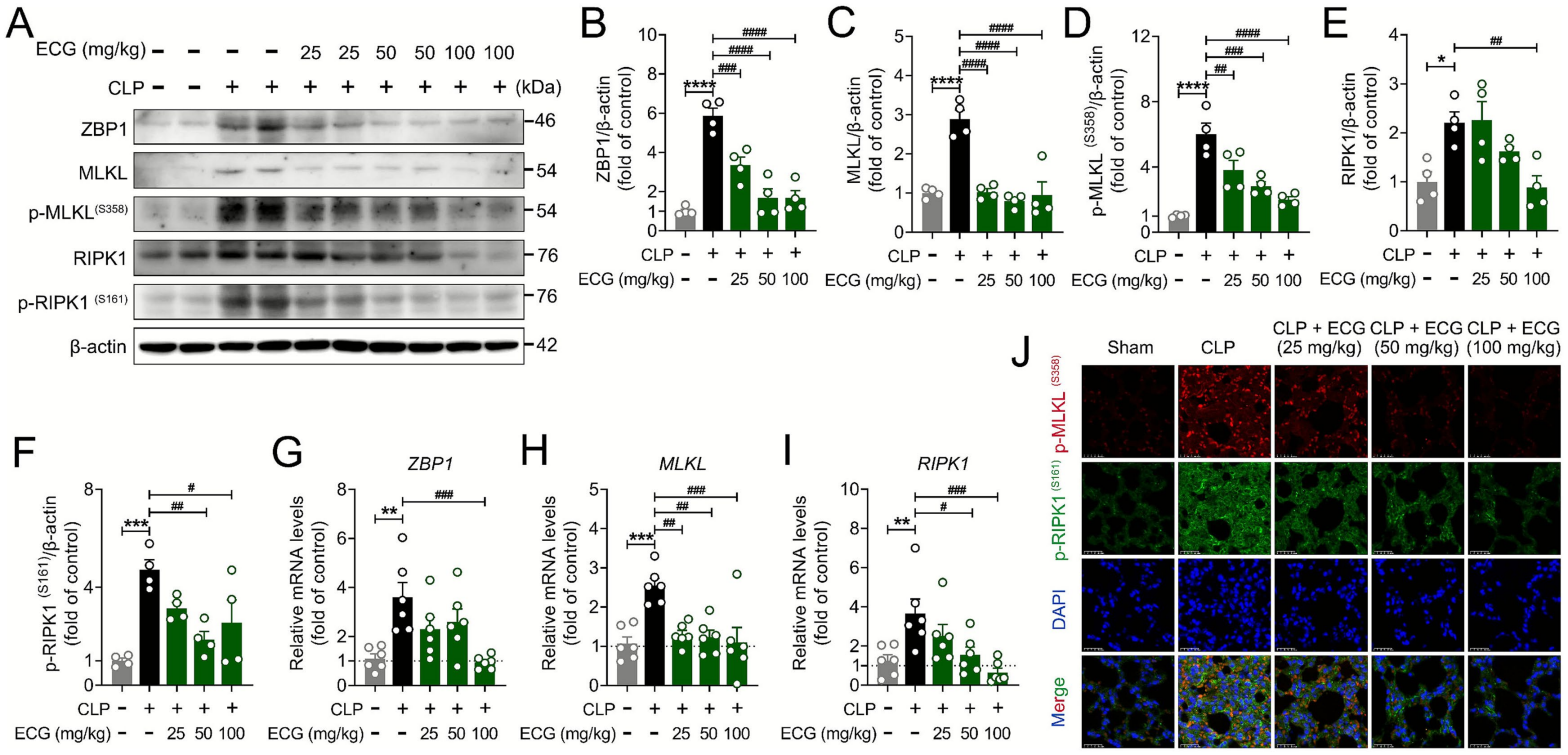
ECG protects sepsis induced ALI mice by inhibiting necroptosis. **(A)** Western blotting analysis of ZBP1, MLKL, p-MLKL, RIPK1, and p-RIPK1 proteins in lung tissues. **(B–F)** Relative intensity of these proteins (*n* = 4). **(G–I)** mRNA level of *ZBP1, MLKL,* and *RIPK1* (*n* = 6). **(J)** Representative immunofluorescence images of p-MLKL, and p-RIPK1-positive in lung tissues. Scale bars: 25 μm. Data are presented as mean ± SEM. Compared to the control group, *p < 0.05, **p < 0.01, ***p < 0.001, and ****p < 0.0001. Compared to the CLP group, ^#^*p* < 0.05, ^##^*p* < 0.01, ^###^*p* < 0.001, and ^####^*p* < 0.0001.

Z-DNA binding protein 1 (ZBP1), as a key molecule in the mechanism of cell death, mediates the assembly of proteins such as RIPK1, CASP8, and ASC into PANoptosis bodies, thereby inducing the occurrence of pan apoptosis (31, 32). We can see from the previous results that ECG effectively improved the occurrence of pyroptosis and apoptosis induced by sepsis in ALI mice. In order to further analyze the molecular mechanism of this process, we conducted further research on key protein signals in necroptosis. Interestingly, Western blot results showed that the ECG treatment group significantly downregulated the expression of pan apoptotic proteins ZBP1, MLKL, p-MLKL, RIPK1, and p-RIPK1 compared to the CLP group (Figures 5A–F), which is consistent with our expected results. At the same time, PCR results showed that the mRNA expression of ZBP1, MLKL, and RIPK1 in the lung tissue of CLP group mice was abnormally increased, and significantly decreased after ECG administration. Notably, this downregulation exhibited a clear dose dependency, with the CLP + ECG (100 mg/kg) group demonstrating the most robust effect (Figures 5G–I). The interaction between p-RIPK1 and RIPK3 forms a necrotic apoptotic complex, which further phosphorylates MLKL, triggering oligomerization and membrane translocation of MLKL, ultimately leading to cell membrane rupture and cell death. Immunofluorescence results showed that p-MLKL and p-RIPK1 proteins were abnormally expressed in the lung tissue of CLP group mice, and their expression significantly decreased after ECG pretreatment. Critically, this reduction was most pronounced in the high-dose ECG group, visually reinforcing the dose-dependent inhibition of necroptosis activation (Figure 5J). In summary, ECG protects sepsis induced ALI mice by inhibiting necroptosis mediated by the ZBP1/MLKL/RIPK1 signaling pathway.

### 3.6 Changes in transcriptome of lung tissue in ALI mice after ECG intervention

**Figure 6:**
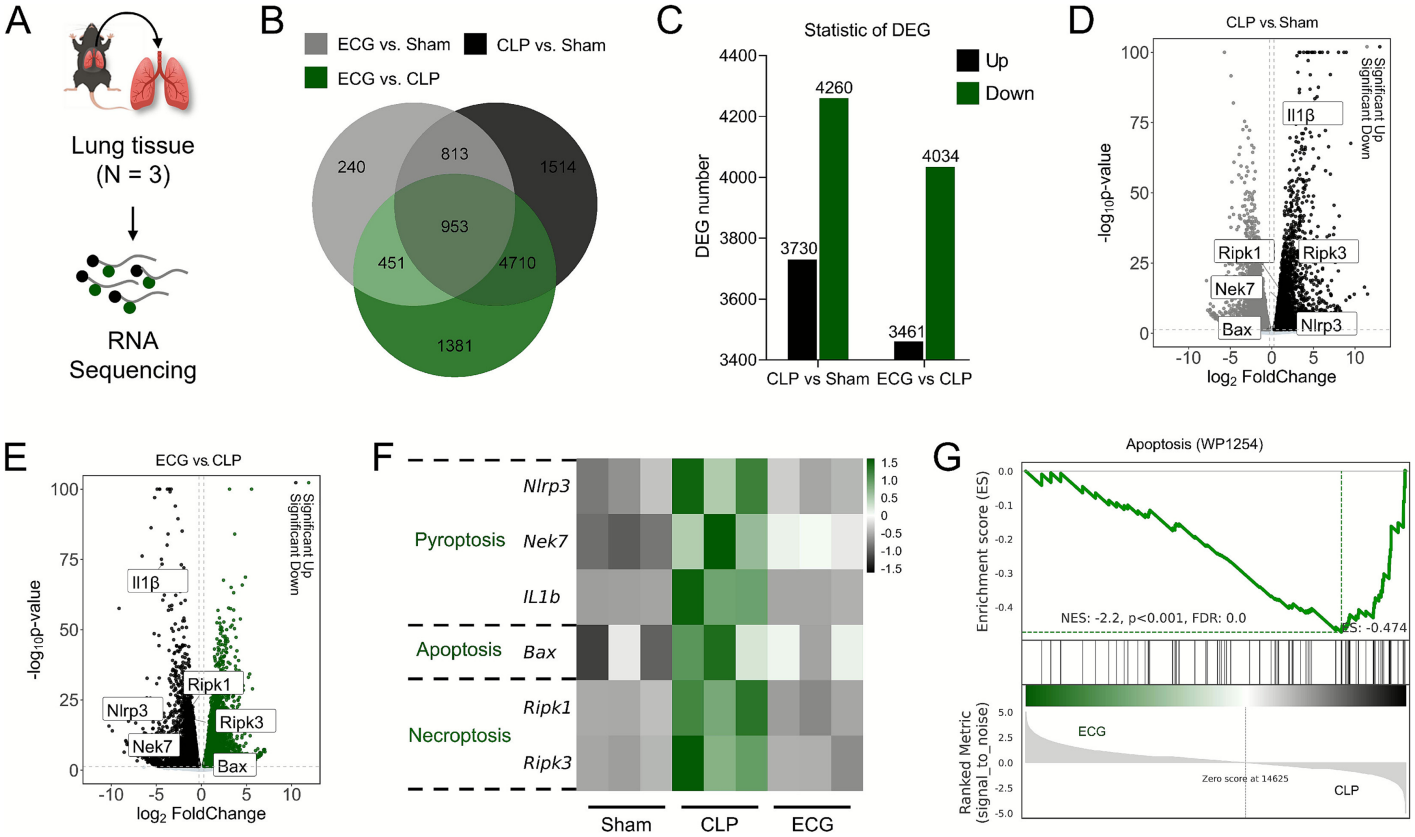
Lung tissue transcriptome changes upon ECG intervention in sepsis induced ALI mice. **(A)** RNA sequencing analysis process (*n* = 3). **(B)** Venn Map. **(C)** Ststistic of DGE. **(D)** CLP vs. Sham comparison volcano plot. **(E)** ECG vs. CLP comparison. **(G)** Heatmap of gene expression related to Pyroptosis, Apoptosis, and Necroptosis. **(H)** GSEA analysis.

In order to more accurately understand the genetic changes of ECG in alleviating sepsis induced ALI, we performed RNA sequence analysis on mouse lung tissue (Figure 6A). The Venn Map results showed that there were 1766 intersecting genes between the Sham group and the CLP group, 5,663 intersecting genes between the CLP group and the ECG group, and 953 common intersecting genes among the three groups (Figure 6B). Meanwhile, compared with the Sham group, there were 7,990 genes with significant DEGs in the CLP group, of which 3,730 genes were up-regulated and 4,260 genes were down regulated. 7,495 DEGs were obtained in the CLP and ECG groups, of which 3,461 genes were significantly up-regulated and 4,034 genes were down regulated (Figure 6C). In addition, volcano plot results showed that compared with the Sham group, IL-1 *β*, Ripk1, Ripk3, Nlrp3, and Nek7 genes were significantly upregulated in the CLP group, while Bax gene expression was significantly downregulated (Figure 6D). Compared with the CLP group, the ECG group significantly downregulated the expression of IL-1 β, Ripk1, Ripk3, Nlrp3, and Nek7 genes, while upregulating the expression of Bax gene (Figure 6E). The relevant heatmap also showed the same results (Figure 6F). Meanwhile, GSEA analysis showed that after ECG intervention, most DEGs involved in cell apoptosis were significantly downregulated (Figure 6G). These bioinformatics analyses are almost consistent with the results obtained from our molecular biology techniques, suggesting that inhibiting the activation and pan apoptotic process of NLRP3 inflammasome is the key to ECG improving sepsis induced ALI.

### 3.7 ECG inhibits LPS induced pyroptosis and necroptosis in RAW264.7 cells

**Figure 7:**
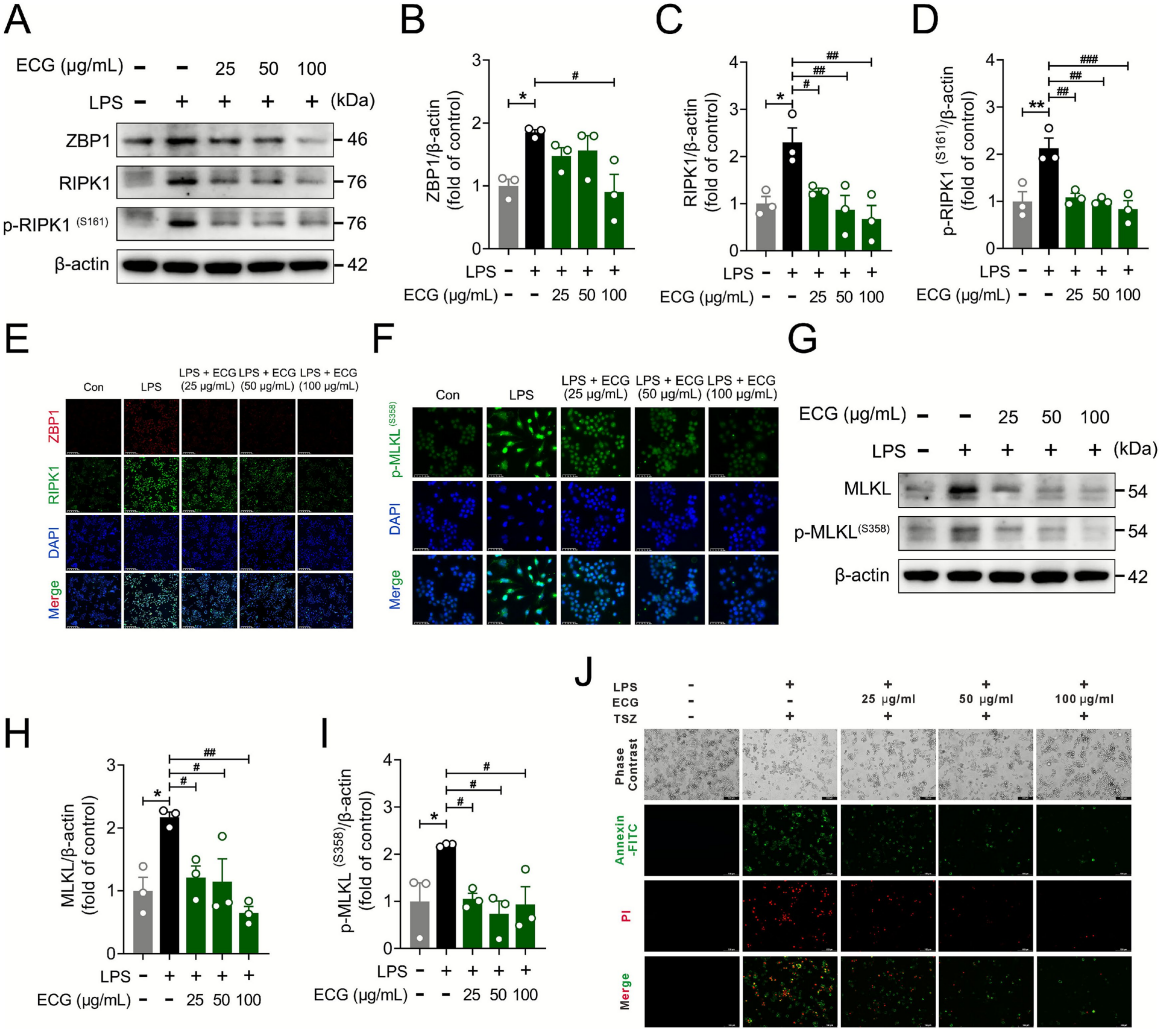
ECG inhibits LPS induced pyroptosis and necroptosis in RAW264.7 cells. **(A)** Western blotting analysis of ZBP1, RIPK1, and p-RIPK1 proteins in cells. **(B–D)** Relative intensity of these proteins (*n* = 3). **(E)** Representative immunofluorescence images of ZBP1- and RIPK1-positive in cells. Scale bars: 25 μm. **(F)** Representative immunofluorescence images of p-MLKL-positive in cells. Scale bars: 25 μm. **(G)** Western blotting analysis of MLKL and p-MLKL proteins in cells. **(H,I)** Relative intensity of these proteins (*n* = 3). **(J)** Representative immunofluorescence images of pyroptosis and necroptosis in cells. Scale bars: 100 μm. Data are presented as mean ± SEM. Compared to the control group, *p < 0.05, **p < 0.01. Compared to the CLP group, ^#^*p* < 0.05, ^##^*p* < 0.01, and ^###^*p* < 0.001.

Our previous study demonstrated ECG’s inhibition of LPS-induced apoptosis and pyroptosis. To further elucidate its role in multiple programmed cell death pathways, we examined ECG’s effects on pyroptosis and necroptosis using an LPS-stimulated RAW264.7 cell model (33, 34). As shown in Figures 7A–D, western blot analysis confirmed that ECG attenuated the LPS-induced increases in ZBP1 and RIPK1 protein levels. Subsequent immunofluorescence analysis revealed that LPS significantly upregulated key necroptosis mediators including ZBP1 and RIPK1, whereas ECG treatment effectively reduced their expression in a concentration-dependent manner, with the most substantial reduction observed at 100 μg/mL (Figure 7E). Regarding necroptosis, immunofluorescence results showed a significant upregulation of p-MLKL protein in LPS-induced cells, which was effectively reversed by ECG treatment (Figure 7F). Similarly, western blot results demonstrated that the protein levels of both MLKL and p-MLKL were increased in LPS-treated cells but were reduced in the ECG-treated groups in a dose-dependent manner (Figures 7G–I). In order to provide multi-faceted evidence for the inhibitory effect of ECG on necroptosis, we subsequently employed two dedicated commercial kits to evaluate both apoptotic and necrotic cell death. Green fluorescence indicates staining with Annexin V-FITC, identifying cells in either apoptosis or necrosis, while red fluorescence corresponds to propidium iodide (PI) staining, which specifically labels necrotic cells. The results demonstrated that the necrosis rate in the ECG-treated groups was significantly lower than that in the model group, with the most pronounced reduction observed in the high-dose group (100 μg/mL) (Figure 7J). These findings indicate that ECG inhibits LPS-induced necroptosis in RAW264.7 cells through the ZBP1/RIPK1/MLKL signaling axis.

### 3.8 ECG components forms stable complexes with ZBP1 and RIPK1

**Figure 8:**
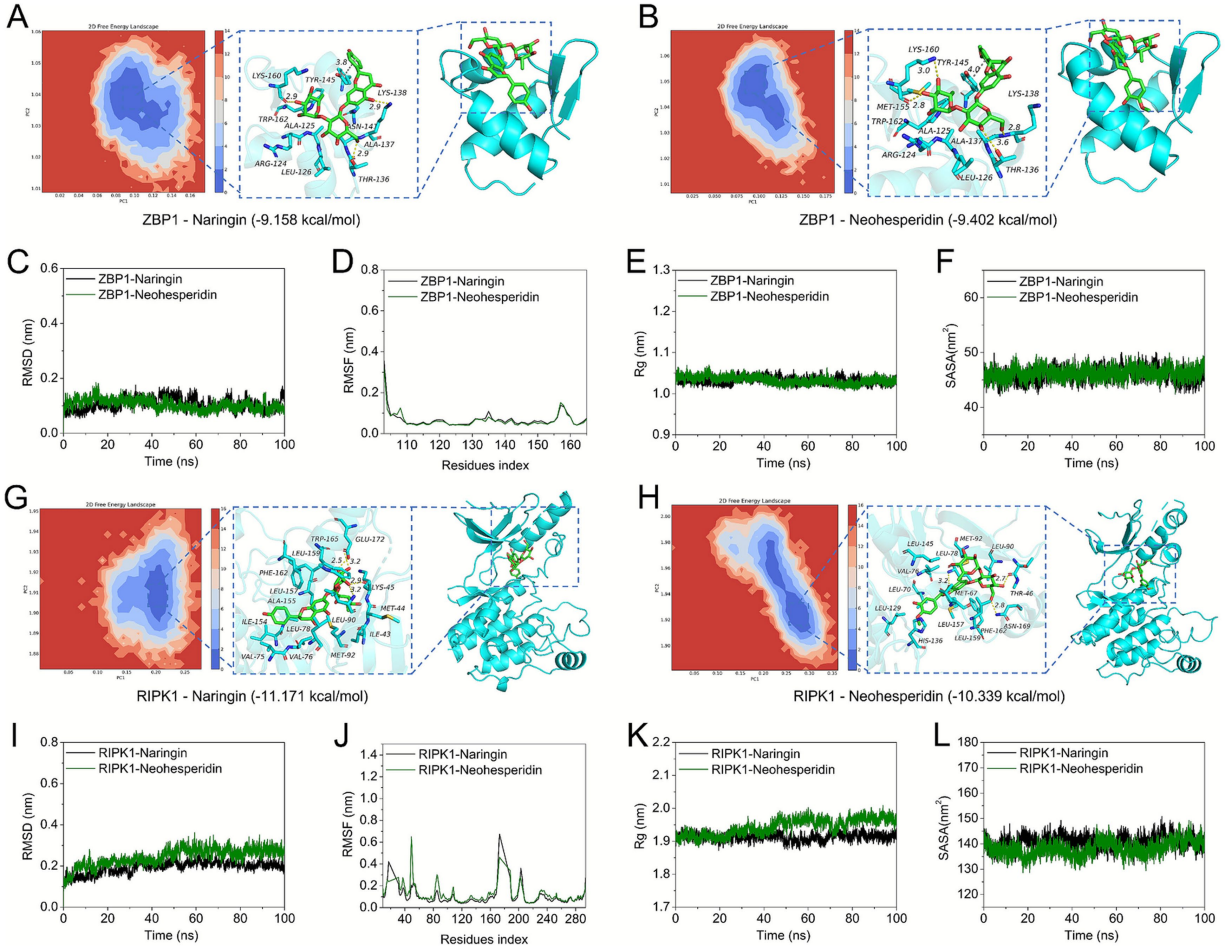
Dynamics simulation analysis demonstrated stable binding of the active components of ECG (Naringin and Neohesperidin) to the PANoptosis-associated targets (ZBP1, RIPK) of ALI. **(A)** The binding mode of ZBP1 with Naringin after 100 ns molecular dynamics. **(B)** The binding mode of ZBP1 with Neohesperidin after 100 ns molecular dynamics. **(C–F)** The RMSD, RMSF, SASA, and Rg of RIPK1 with Naringin, Neohesperidin. **(G)** The binding mode of RIPK1 with Naringin after 100 ns molecular dynamics. **(H)** The binding mode of RIPK1 with Neohesperidin after 100 ns molecular dynamics. **(I–L)** The RMSD, RMSF, SASA, and Rg of RIPK1 with Naringin, Neohesperidin.

MD simulations were employed to validate the binding stability of bioactive compounds with target proteins, as this approach provides critical insights into intermolecular interactions and complex stability. Neohesperidin formed hydrogen bonds with key ZBP1 residues (LYS-160, THR-136, LYS-138) and hydrophobic interactions with LEU-126, ALA-125, MET-155, TRP-162, TYR-145, and ALA-137, with its phenyl ring establishing *π*-π stacking with Tyr-145, while Naringin similarly engaged ZBP1 through hydrogen bonding, hydrophobic, and conjugated interactions (Figures 8A,B). Both Naringin-ZBP1 and Neohesperidin-ZBP1 complexes exhibited average RMSD values below 2 Å (Figure 8C), with marginally lower RMSD in Neohesperidin-ZBP1 suggesting slightly enhanced stability. Although minor conformational fluctuations occurred in some amino acid residues (Figure 8D), most maintained changes within acceptable limits. Decreased Rg values (Figure 8E) and stable solvent-accessible surface areas (SASA) near 48 nm^2^ (Figure 8F) further confirmed binding robustness. For RIPK1, Naringin formed hydrogen bonds with Leu-90, Glu-172, Lys-45 and hydrophobic contacts with VAL-75, ILE-154, VAL-76, MET-92, LEU-78, ALA-155, LEU-157, PHE-162, ILE-43, MET-44, and TRP-165, with Neohesperidin establishing analogous interactions (Figures 8G,H). Both RIPK1 complexes showed RMSD < 3 Å (Figure 8I), with most residues exhibiting acceptable conformational flexibility (Figure 8J). While Neohesperidin-RIPK1 displayed moderate Rg increases, Naringin-RIPK1 maintained stable Rg (Figure 8K), and consistent SASA values near 140 nm^2^ (Figure 8L) confirmed effective binding. The ΔGbinding energy values for these molecule-protein complexes were provided in Supplementary Table 3. These results demonstrate stable binding of ECG’s components including Neohesperidin and Naringin to PANoptosis targets including ZBP1 and RIPK1 through multifaceted molecular interactions.

## 4 Discussion

Sepsis-induced ALI remains a serious clinical threat to this day, primarily due to its complex pathogenesis and the current lack of effective treatment strategies. Research has shown that the pathological process of sepsis involves multiple factors, with dysregulated host defense responses and the subsequent excessive systemic inflammatory reaction being particularly critical (35). Additionally, mitochondrial dysfunction and oxidative stress play significant roles in driving disease progression. Among the various affected organs, the lungs are often the earliest and most susceptible target of sepsis-induced damage (36, 37). Clinically, sepsis-induced ALI/acute respiratory distress syndrome (ARDS) is associated with an extremely high mortality rate and has become one of the leading causes of death in critically ill patients (38, 39). Therefore, elucidating the molecular biological mechanisms underlying the development and progression of sepsis-induced lung injury holds great theoretical significance and clinical value.

The high mortality associated with sepsis-induced ALI is reliably recapitulated in the CLP model, which mirrors the progressive hyper-inflammation and multi-organ dysfunction observed in clinical sepsis. This model typically exhibits substantial mortality, primarily driven by uncontrolled systemic inflammation and extensive cellular damage (40, 41). Consistent with this paradigm, our study recorded a 80% mortality rate in the CLP group within the observation period. Crucially, prophylactic treatment with ECG dose-dependently improved survival, with the high-dose regimen achieving a significant survival rate of 70%. This marked enhancement in survival aligns with our mechanistic findings, suggesting that the improved outcome is likely attributable to the attenuation of PANoptosis and the associated inflammatory cascade. Therefore, the survival benefit conferred by ECG not only underscores its therapeutic potential but also strongly justifies further investigation into the detailed molecular circuitry through which it coordinates this multi-faceted protection against sepsis-induced lethality.

Existing evidence indicates that PANoptosis can directly induce the death of alveolar epithelial cells and vascular endothelial cells, thereby compromising the structural integrity of lung tissue (42). Although previous studies have confirmed the critical role of PANoptosis in the pathogenesis of various inflammatory diseases, its specific function and regulatory mechanisms in sepsis-induced ALI remain incompletely understood (43, 44). In contrast, through systematic *in vivo* and *in vitro* validation, our study demonstrates that ECG effectively suppresses the activation of PANoptosis, thereby exerting significant protective effects against sepsis-induced ALI. Our findings indicate that ECG mitigates systemic and pulmonary inflammation, as evidenced by reduced levels of IL-1β, IL-6, and TNF-*α* in both serum and bronchoalveolar lavage fluid. These anti-inflammatory effects were accompanied by attenuated lung histopathological injury, including decreased edema and inflammatory cell infiltration. Importantly, ECG downregulated key mediators of the NLRP3/Caspase-1/GSDMD pyroptosis pathway and the Bcl-2/Bax/Caspase-3 apoptosis axis, consistent with earlier reports on its role in LPS-induced ALI (45). Moreover, we identified a novel mechanism by which ECG inhibits ZBP1/MLKL/RIPK1-driven necroptosis, thereby contributing to the attenuation of PANoptosis. The convergence of multiple cell death pathways in sepsis has gained increasing attention. PANoptosis represents an integrated cell death modality that exacerbates tissue damage and inflammatory amplification. Our data suggest that ECG interferes with this process at multiple nodes, likely through its bioactive components such as naringin and neohesperidin, which we confirmed via molecular dynamics simulations to stably interact with ZBP1 and RIPK1. These interactions may disrupt the formation of the necrosome complex and subsequent MLKL activation, thereby preserving pulmonary microvascular integrity.

Despite the promising findings, this study has several limitations that should be acknowledged. The translational relevance of our results, though derived from a CLP model that closely recapitulates human sepsis, requires further validation in additional experimental models or species. Furthermore, while naringin and neohesperidin were identified as key bioactive compounds, the potential contributions and synergistic effects of other constituents within the complex mixture of ECG remain unexplored. The precise intracellular signaling events that link ECG to the inhibition of PANoptosis also warrant deeper investigation. Future research should leverage advanced techniques such as single-cell RNA sequencing and spatial proteomics to precisely map the cellular targets of ECG within the lung microenvironment and elucidate its upstream regulatory mechanisms. Additionally, given the critical role of neutrophils in sepsis-induced ALI, the impact of ECG on immune cell populations, particularly neutrophil recruitment and activation, represents a crucial direction for further inquiry. Finally, preclinical trials evaluating ECG in combination with standard sepsis therapies are needed to assess its potential and feasibility as an adjunctive treatment.

## 5 Conclusion

Our research results demonstrate that ECG alleviates sepsis-induced ALI by inhibiting the NLRP3/Caspase-1/GSDMD signaling pathway-mediated pyroptosis, the Bcl-2/Bax/Caspase-3 signaling pathway-mediated apoptosis, and regulating the ZBP1/MLKL/RIPK1 signaling pathway-mediated necroptosis. These findings establish a robust scientific foundation for the development of ECG-based functional foods and their application as dietary interventions for sepsis-induced ALI.

## Data Availability

The original contributions presented in the study are included in the article/Supplementary material, further inquiries can be directed to the corresponding author/s.
