# Supplementary material for: Protective effects of Exocarpium Citri Grandis against sepsis-induced acute lung injury via PANoptosis inhibition
Source: Front Nutr. 2025 Dec 22;12:1661404. doi: 10.3389/fnut.2025.1661404 (PMC12766971; doi:10.3389/fnut.2025.1661404)
Supplement: Supplementary file 1 [file Table_1.pdf]

Identification of 15 Compounds in ECG

Supplementary Material Methods: An Agilent 1290 infinity UHPLC system (California, U.S.A.) with a binary pump, an autosampler, and a thermostated column compartment coupled with 6545XT Q-TOF-MS system (California, U.S.A.) was used for the identification of ECG. They were separated using an Agilent ZORBAX Eclipse Plus C18 column with length of 2.1 × 100 mm, 1.8 μm particle size (Agilent, California, U.S.A.). The mobile phase included phase A, containing Distillation-Distillation H2O with 0.1% FA, and phase B, which was ACN with 0.1% FA. The linear gradient elution was maintained as follows: 5% B in 0-0.5 min, 5%-10% B in 0.5-3 min, 10%-30% B in 3-10 min, 30%-60% B in 10-15 min, 60%-100% B in 15-18 min, 100% B in 18-21 min, 100%-5% B in 21-21.1 min, flowing at 0.35 mL/min. The column temperature was operated at a temperature of 35 ℃ with an injection volume of 2 μL. The temperatures of dry gas and sheath gas were set at 320 and 400°C, with respective flow rates of 10 and 12 L/min. The nebulizer operated at 45 psi, with the capillary and nozzle voltages set to 4000 V and 500 V, respectively, for positive ESI mode. The fragmentor was adjusted to 180 V, and the collision energy was set to 30 eV. Mass spectrometry data were gathered in centroid mode across an m/z range of 100-1700. Finally, all data were processed using Agilent MassHunter Qualitative Analysis B.10.

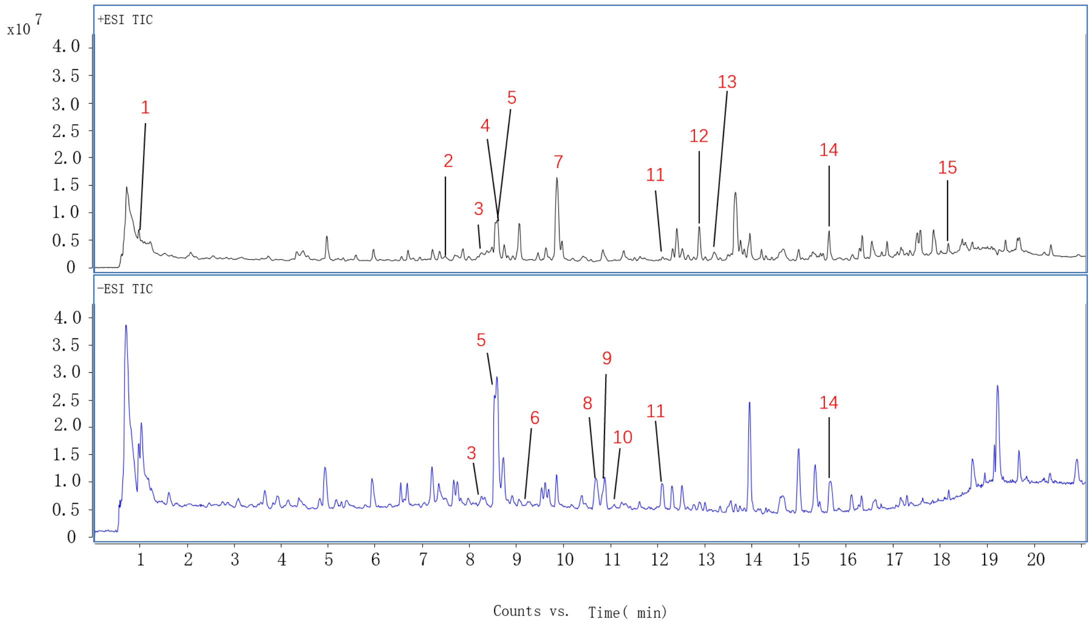

Supplementary Figure 1. Overview of 15 compounds of ECG identified by UHPLC-Q-Exactive analysis. Compounds were identified as Stachydrine (1), Cnidioside a (2), Narirutin (3), Naringin (4), Neohesperidin (5), Dihydrokaempferol (6), Poncirin (7), Bergaptol (8), Naringenin chalcone (9), Apigenin (10), Marmin (11), Bergapten (12), Isomerazin (13), Isoimperatorin (14), and Auraptene (15)

Supplementary Table 1. UPLC-Q/TOF-MS/MS Analysis Table of 15 Chemical Compounds in ECG.

No.	Components	Formula	RT min	ESI	m/z theoretical value	m/z actual value	ppm	MS/MS

1	Stachydrine	$C_7H_{13}NO_2$	0.872	[M+H]+	144.1019	144.1024	3.47	58.0651, 84.0805
2	Cnidioside a	$C_{17}H_{20}O_9$	7.446	[M+NH4]+	386.1446	386.1441	-1.29	85.0276, 147.0441, 189.0547, 207.0642
3	Narirutin	$C_{27}H_{32}O_{14}$	8.251	[M-H]-	579.1719	579.1714	-0.86	151.0042, 271.0619
	Narirutin	$C_{27}H_{32}O_{14}$	8.265	[M+H]+	581.1865	581.1862	-0.52	71.0487, 85.0282, 119.0496, 153.0165, 273.0751, 401.1173
4	Naringenin chalcone	$C_{15}H_{12}O_5$	8.529	[M+H]+	273.0757	273.0753	-1.46	119.0485, 147.0438, 153.0187, 154.0210, 255.0661
5	Naringin	$C_{27}H_{32}O_{14}$	8.569	[M+H]+	581.1865	581.1862	-0.52	71.0491, 85.0286, 121.0651, 153.01081, 129.0543, 195.0291, 273.0767, 339.0866, 383.1135, 401.1227
	Naringin	$C_{27}H_{32}O_{14}$	8.577	[M-H]-	579.1719	579.1714	-0.86	119.0494, 151.0036, 271.0605
6	Dihydrokaempferol	$C_{15}H_{12}O_6$	9.199	[M-H]-	287.0561	287.0556	-1.74	125.0249, 177.0572, 259.0590
7	Isomerazin	$C_{15}H_{16}O_4$	9.880	[M+H]+	261.1121	261.1117	-1.53	103.0543, 131.0493, 159.0433, 189.0552, 201.0542, 243.1009
8	Bergaptol	$C_{11}H_6O_4$	10.666	[M-H]-	201.0193	201.0188	-2.49	117.0344, 129.0340, 145.0292, 157.0274, 173.0223
9	Neohesperidin	$C_{28}H_{34}O_{15}$	10.878	[M-H]-	609.1825	609.1816	-1.48	255.0663, 256.0692, 301.0708, 563.1776, 564.1800
10	Poncirin	$C_{28}H_{34}O_{14}$	11.075	[M-H]-	593.1876	593.1865	-1.85	83.0130, 285.0768
11	Apigenin	$C_{15}H_{10}O_5$	12.121	[M-H]-	269.0455	269.0454	-0.37	119.0506, 151.0037, 177.0183, 227.0711, 253.0507
	Apigenin	$C_{15}H_{10}O_5$	12.139	[M+H]+	271.0601	271.0598	-1.11	121.0655, 147.0437, 153.0174
12	Marmin	$C_{19}H_{24}O_5$	12.880	[M+H]+	333.1697	333.1692	-1.50	107.0584, 135.1166, 163.0389
13	Bergapten	$C_{12}H_8O_4$	13.197	[M+H]+	217.0495	217.0492	-1.38	173.0587, 202.0257, 203.0286
14	Isoimperatorin	$C_{16}H_{14}O_4$	15.681	[M-H]-	269.0819	269.0823	1.49	254.0598, 201.0184

	Isoimperatorin	$C_{16}H_{14}O_4$	15.691	[M+H]+	271.0965	271.0964	-0.37	203.0331, 175.0387, 147.0438, 131.0469
15	Auraptene	$C_{19}H_{22}O_3$	18.179	[M+H]+	299.1642	299.1645	1.00	69.0697, 81.0695, 95.0851, 137.1324, 163.0390, 164.0426, 189.0517

Supplementary Table 2. Primers used in qPCR analysis.

Gene	Forward Primer	Reward Primer
IL1β	CCGTGGACCTTCCAGGATGA	GGGAACGTCACACACCAGCA
IL6	TAGTCCTTCCTACCCCAATTTCC	TTGGTCCTTAGCCACTCCTTC
Tnfα	CCCTCACACTCAGATCATCTTCT	GCTACGACGTGGGCTACAG
Ccl2	TTAAAAACCTGGATCGGAACCAA	GCATTAGCTTCAGATTTACGGGT
Ccl3	TTCTCTGTACCATGACACTCTGC	CGTGGAATCTTCCGGCTGTAG
Ccl4	TTCCTGCTGTTTCTCTTACACCT	CTGTCTGCCTCTTTTGGTCAG
Ccl5	TCGAGTGACAAACACGACTGC	GCTGCTTTGCCTACCTCTCC
Ccl7	GCTGCTTTCAGCATCCAAGTG	CCAGGGACACCGACTACTG
Cxcl9	TCCTTTTGGGCATCATCTTCC	TTTGTAGTGGATCGTGCCTCG
NLRP3	TGTGAGAAGCAGGTTCTACTCT	TGTAGCGACTGTTGAGGTCCA
ASC	CTTGTCAGGGGATGAACTCAAAA	GCCATACGACTCCAGATAGTAGC
Bax	TGAAGACAGGGGCCTTTTTG	AATTCGCCGGAGACACTCG
Bcl2	GACAAGGAGATGCAGGTATTGG	TCCCGTAGAGATCCACAAAAGT
ZBP1	AAGAGTCCCCTGCGATTATTTG	TCTGGATGGCGTTTGAATTGG
MLKL	AATTGTACTCTGGGAAATTGCCA	TCTCCAAGATTCCGTCCACAG
RIPK1	GAAGACAGACCTAGACAGCGG	CCAGTAGCTTCACCACTCGAC
β-actin	GGCTGTATTCCCCTCCATCG	CCAGTTGGTAACAATGCCATGT

Supplementary Table 3. The binding energy by MMGBSA (kcal/mol).

Type	ZBP1-Naringin	ZBP1-Neohesperidin	RIPK1-Naringin	RIPK1-Neohesperidin
E_{VDW}	-28.9+/-0.25	-33.56+/-0.37	-69.78+/-1.97	-71.57+/-0.34
E_{ELE}	-21.69+/-6.31	-21.04+/-3.22	-58.64+/-3.35	-27.2+/-6.39
E_{GB}	36.5+/-1.78	40.37+/-1.35	90.06+/-1.23	66.36+/-2.13
E_{SA}	-3.4+/-0.05	-3.69+/-0.04	-5.94+/-0.02	-6.15+/-0.04
$G_{binding\ energy}$	-17.49+/-6.56	-17.92+/-3.51	-44.31+/-4.08	-38.56+/-6.74

E_{VDW}: van der Waals energy

E_{ELE}: eletrostatic energy

E_{GB}: polar contribution to solvation

E_{SA}: non-polar contribution to solvation

Supplementary GO enrichment analysis: The GO enrichment analysis of the top 30 terms revealed a highly coordinated cellular program centered on inflammatory response processes. In Biological Processes (BP), the most significant findings were the upregulation of terms related to chemokine-mediated signaling pathway and the cellular response to lipopolysaccharide, which directly drove a robust inflammatory response. This was coupled with a striking activation of processes associated with cell motility, including the positive regulation of smooth muscle cell proliferation, cell migration, and, most notably, a suite of functions pertaining to ciliary movement and cilium assembly. This suggests that the inflammatory trigger is closely linked to the enhanced assembly and beating of motile cilium. The Cellular Component (CC) analysis robustly supported this interpretation. The results were overwhelmingly localized to the cytoplasm, cell projection, and specifically to the cilium and axoneme, which is the core cytoskeletal structure of a motile cilium characterized by a "9+2" microtubule arrangement. Additionally, components of the extracellular space, region, and extracellular matrix were enriched, indicating active communication between the cell and its environment. Finally, Molecular Function (MF) terms detailed the mechanistic basis for these processes. The cells exhibited increased capacity for transmembrane signaling receptor activity and chemokine/growth factor/cytokine activity, underpinning the inflammatory and proliferative signals. These functions were supported by specific binding capabilities, including heparin binding, calcium ion binding, and protein homodimerization. Crucially, the presence of minus-end-directed microtubule motor activity confirms the functional activation of dynein motors responsible for the power generation of ciliary beating. In summary, the GO analysis paints a coherent picture of an inflammatory state that is intrinsically linked to the activation of motile cilia assembly and function, potentially aimed at enhancing cellular movement or fluid flow in response to pathogenic or inflammatory stimuli.

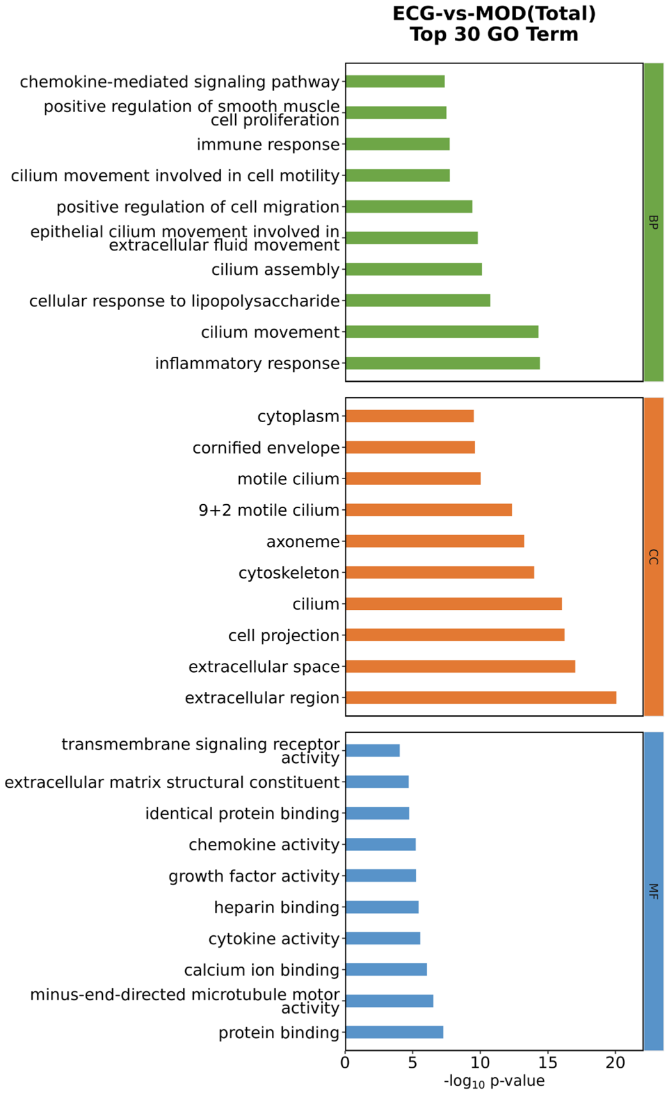

Supplementary Figure 2. GO enrichment analysis (CLP vs ECG).

Supplementary KEGG enrichment analysis: According to the KEGG pathway enrichment analysis, we found that the compound ECG is involved in regulating apoptosis and necroptosis pathways. In the pathway map, targets are color-coded based on their response to ECG treatment: blue indicates downregulated targets, red indicates upregulated targets, light green represents related targets within the pathway that were not significantly regulated by ECG, and dark green reflects bidirectional regulatory effects, where ECG upregulates some targets while downregulating others in the same pathway. These results suggest that ECG may modulate cell death-related signaling through multiple targets and mechanisms.

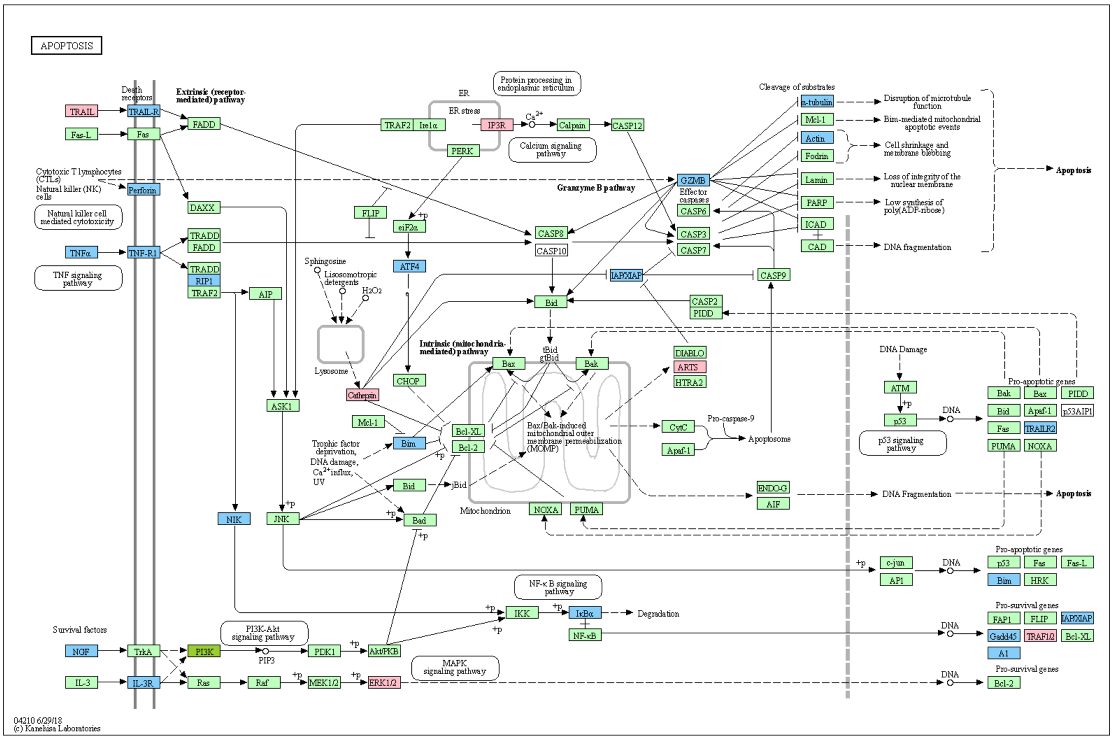

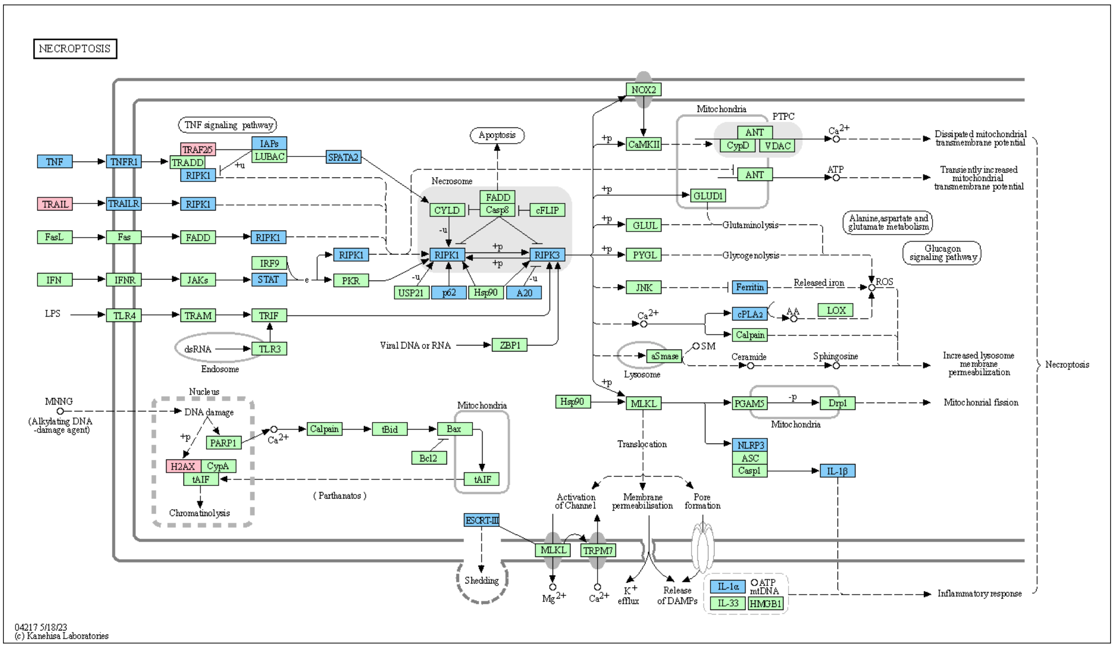

Supplementary Figure 3. KEGG enrichment analysis (CLP vs ECG). Blue indicates downregulated targets, red indicates upregulated targets, light green represents related targets within the pathway that were not significantly regulated by ECG, and dark green reflects bidirectional regulatory effects.
